# Seroprevalence of IgG Antibodies to Measles, Mumps, Rubella and Varicella in Adolescents with Documented Two-Dose MMR Vaccination: Cross-Sectional Study

**DOI:** 10.3390/antib15050082

**Published:** 2026-09-04

**Authors:** Mikhail P. Kostinov, Ivan S. Samolygo, Aristitsa M. Kostinova, Natalya L. Starikova, Marina A. Manina, Valentina B. Polishchuk, Albina S. Pestova, Pavel I. Zhuravlev, Anton S. Antishin, Svetlana I. Erdes

**Affiliations:** 1 Department of Epidemiology and Modern Vaccination Technologies, Institute of Professional Education, I.M. Sechenov First Moscow State Medical University (Sechenov University), Moscow 105064, Russia; monolit.96@mail.ru (M.P.K.); aristica_kostino@mail.ru (A.M.K.); 2 Laboratory of Vaccine Prevention and Immunotherapy of Allergic Diseases, Federal State Budgetary Scientific Institution, I. Mechnikov Research Institute of Vaccines and Sera, Moscow 105064, Russia; polischook@mail.ru (V.B.P.); pvazhurik@gmail.com (P.I.Z.); 3 Department of Propaedeutics of Children’s Diseases, N.F. Filatov Clinical Institute of Children’s Health, I.M. Sechenov First Moscow State Medical University (Sechenov University), Moscow 119991, Russia; manina_m_a@staff.sechenov.ru (M.A.M.); pestova_a_s@staff.sechenov.ru (A.S.P.); antishin_a_s@staff.sechenov.ru (A.S.A.); erdes_s_i@staff.sechenov.ru (S.I.E.); 4 L.I. Sokolova Yoshkar-Ola Children’s City Hospital, Yoshkar-Ola 424000, Russia; starikova.72@inbox.ru

**Keywords:** measles, mumps, rubella, varicella, vaccine-induced immunity, adolescents

## Abstract

**Background**: Measles, mumps, and rubella outbreaks continue to occur despite high vaccination coverage, raising concerns about long-term vaccine-induced immunity. This study assessed serological protection against measles, mumps, rubella, and varicella in fully vaccinated Russian adolescents to identify immunity gaps and inform revaccination strategies. **Methods**: Serum IgG was quantified by commercial ELISA. Analytical cut-offs were: measles ≥ 0.18 IU/mL, rubella ≥ 30 IU/mL, mumps index ≥ 1.0 (index = OD/0.467), and varicella ≥ 1.0 IU/mL; equivocal results were classified as negative in the primary analysis and as positive in a sensitivity analysis. Geometric mean concentrations (GMC) were computed on the natural-logarithm scale. **Results**: Seropositivity differed markedly between infections (χ^2^ 55.1; *p* < 0.001): rubella 81.4% (95% CI 74.6–86.7), varicella 57.7% (95% CI 49.8–65.2), mumps 53.2% (95% CI 45.4–60.9) and measles 41.0% (95% CI 33.6–48.9). GMC was lowest for measles (0.132; 95% CI 0.111–0.158) and highest for rubella (61.9; 95% CI 55.3–69.2). The measles estimate was sensitive to the treatment of equivocal results, rising to 53.2% (95% CI 45.4–60.9) at the manufacturer’s cut-off of ≥0.12 IU/mL; rubella rose to 94.9% at ≥16 IU/mL. **Conclusions**: Lower seropositivity rates for measles and mumps were identified among fully vaccinated adolescents, which fall below standard epidemiological benchmarks. In contrast, rubella seropositivity was robust at 81.4%. Because the measles estimate depended strongly on the assay cut-off applied, and because enzyme immunoassays are known to underestimate seroprevalence relative to neutralization testing, these findings describe the distribution of circulating antibody rather than the prevalence of susceptibility and warrant confirmation in larger representative samples with neutralization testing.

## 1. Introduction

Measles, rubella, mumps, and varicella are vaccine-preventable infectious diseases whose spread can be prevented through specific preventive measures, including vaccination [1,2,3,4]. The accumulated experience with combined measles, mumps, and rubella (MMR) vaccines has demonstrated their high immunogenicity and low reactogenicity [2,5]. Similar data have been obtained for varicella vaccines [6]. However, evidence suggests that 5 to 10% of individuals vaccinated with various vaccines against these infections remain seronegative after primary vaccination, and booster doses do not always provide adequate protection [7].

Despite high MMR vaccination coverage (96–98%) in Russia [8], the proportion of adolescents maintaining an immune response over time remains below desirable levels. It has been shown that unvaccinated individuals, as well as those with unknown vaccination history, account for 46% to 70% of measles cases, while up to 30% of all cases occur in those who received one dose of measles vaccine [8]. According to official statistics, in 2025, the number of immunized children against measles in Russia exceeded 97%, which is a satisfactory indicator (with a target value of 95%). However, the proportion of individuals receiving measles revaccination (children aged 6 years and older) did not reach the target level and was less than 90% [8]. There is a large proportion of individuals whose vaccination history is unknown for various reasons: loss of vaccination certificates, among others. The highest number of measles cases occurs in children aged 1 to 9 years, as well as in adults under 35 years of age [8,9]. Thus, according to official Russian statistics, in 2025, the highest measles incidence rates were observed among children under 1 year of age (47.4 per 100,000 population), and the epidemic process was sustained by unvaccinated individuals and those with unknown vaccination history—90.8% of cases [8]. Furthermore, a negative trend contributing to the spread of the epidemic process (this statement applies to all vaccines in question) is the growing anti-vaccination sentiment among parents who deliberately refuse to vaccinate their children in early childhood and preschool age [10,11]. Researchers identify the main reasons for vaccine refusal as fear of complications and diseases following the procedure, distrust in vaccine quality, personal beliefs, and others [10].

The epidemiological situation regarding rubella remains one of the most favorable, especially in countries with developed healthcare systems [12]. Japan represents one of the most illustrative examples of combating rubella transmission [13]. However, this immunization strategy had its flaws, which led to corresponding consequences: increases in rubella incidence were observed in 2012–2013 and 2018–2019 [10]. Since 2017, the Russian Federation has been declared a rubella-free country. However, according to recent data, in 2025, an increase in rubella incidence was recorded (more than 374 cases nationwide), and cases of severe congenital rubella were also documented [8]. At the same time, rubella vaccination coverage, as with measles, was satisfactory for children in their first year of life, but the revaccination of children over 6 years of age did not reach adequate levels [8].

The global epidemiological situation regarding mumps remains as follows: in the 21st century, several major increases in mumps incidence were identified in 2004–2009 and 2016–2020 [4]. Regional analysis showed higher incidence rates in North and South America (29.2%) and the Eastern Mediterranean region (28.8%) compared to Europe (7.6%) and Southeast Asia (9.6%) [4]. Several major mumps outbreaks were registered in Russia, particularly in 2017–2019 and 2023–2025 [8]. According to official statistics, 1673 mumps cases were registered in Russia in 2025, with notable regional differences (the peak of the epidemic process occurred in the North Caucasus region) [8]. The main sources of the epidemic process were either unvaccinated individuals or those with unknown vaccination history, accounting for 83.6% of all cases. Notably, those vaccinated once contracted the disease in 6% of cases; those vaccinated twice in 10.5% of cases. Timely immunization coverage of children over one year of age against mumps reached target values (95%); however, this indicator did not reach target values for revaccination conducted at ages over 6 years [8].

Varicella incidence rates among children are tens of times higher than among adults (2660 per 100,000 child population) [8]. The majority of cases (49.7%) were observed in the 3–6 years age group [8]. It should be noted separately that varicella vaccination is not included in the National Immunization Schedule in Russia (not covered by insurance), and its administration is a personal decision of each parent [14]. Vaccination of children with MMR is carried out in two stages: children receive the first dose at 12 months of age and the second dose at 6–7 years of age [14].

It is well known that the degree of immune response is genetically determined and largely depends on the child’s health status. In recent years, there has been an increase in the number of children and adolescents with frequent and prolonged respiratory infections, as well as concomitant allergic diseases and/or chronic or latent infections, which are sometimes considered contraindications to vaccination at the recommended age. This situation affects herd immunity against vaccine-preventable infections and leads to its decline, which can trigger localized outbreaks [15,16].

Given the unfavorable epidemiological situation with measles and mumps, as well as the absence of varicella vaccination in the National Immunization Schedule in Russia, we considered it important and necessary to assess the seroprevalence of antibodies to measles, mumps, rubella, and varicella in adolescents aged 13–15 years following routine MMR vaccination, as well as the seroprevalence of varicella antibodies acquired after natural infection, which was the aim of our study.

## 2. Materials and Methods

This cross-sectional study included adolescents aged 13–15 years. Patients were enrolled in the study during the period from January 2025 to June 2025.

Inclusion criteria:

- Documented history of two-dose vaccination against measles, mumps, and rubella;
- Documented history of varicella infection;
- Age of participants from 13 to 15 years inclusive at the time of blood sampling;
- Absence of known primary and secondary immunodeficiency states, oncological, autoimmune, autoinflammatory diseases, or ongoing immunosuppressive therapy before or at the time of study enrollment;
- No blood transfusion and/or human immunoglobulin administration in the previous 12 months;
- Provision of informed consent from study participants and their parents/guardians.

Exclusion criteria:

- History of documented clinical infection caused by measles, mumps, or rubella viruses;
- Acute infectious viral or bacterial infection at the time of study enrollment;
- Incomplete data or absence of data on vaccination history;
- Absence of signed informed consent from study participants.

The study was conducted in accordance with the Declaration of Helsinki and approved by the Ethics Committee of the Ulyanovsk State University (Protocol No. 214, dated 12 January 2025). Demographic data, including age, sex, and health status classification, were obtained through structured interviews with parents or guardians, as well as with the study participants themselves, and through analysis of medical documentation. Health status was classified according to standardized pediatric health classification criteria used in routine clinical practice [17]:

- Group I: clinically healthy children without chronic diseases or functional abnormalities (e.g., normal physical and neuropsychological development, with no history of chronic diseases or organ pathology).
- Group II: children with minor functional abnormalities not requiring continuous medical supervision (e.g., mild postural abnormalities, vegetative dysfunction, mild seasonal allergies in stable remission, and frequent acute respiratory infections without chronic organ pathology).
- Group III: children with chronic diseases in remission or compensated state requiring periodic medical supervision (e.g., controlled bronchial asthma, chronic gastritis in stable remission, and compensated biliary dyskinesia).

Vaccination data were obtained from immunization records. Time elapsed since last vaccination was calculated as the interval in months between the last documented vaccine dose and the date of serological analysis.

Study material. Blood was collected from study participants in disposable plastic tubes with clotting activator. Samples were allowed to clot at room temperature for 15 min, then centrifuged at 3000 rpm for ten minutes to separate serum. Serum aliquots were stored under specific conditions at −70 °C until analysis for no more than 3 months. Patient samples were analyzed in the Laboratory of Vaccine Prevention and Immunotherapy of Allergic Diseases of the Institute using certified equipment from the Core Facility Center of the Mechnikov Research Institute of Vaccines and Sera. IgG antibody levels to measles, rubella, mumps, and varicella in serum samples were determined by solid-phase ELISA using the “Vector-Best IgG-measles” test system (Novosibirsk, Russia) according to the manufacturer’s instructions. According to the accompanying regulatory-technical documentation for quantitative determination of IgG antibodies to measles virus, the test result was considered negative if the antibody concentration in the tested sample was less than 0.11 IU/mL and positive if the antibody concentration was greater than or equal to 0.18 IU/mL. Sera with borderline IgG antibody concentrations to measles virus in the range of 0.12–0.17 IU/mL were classified as negative, since this antibody level cannot be considered fully protective against disease development [18]. Similarly, rubella antibody levels were assessed by solid-phase ELISA using the “Vector-Best IgG-rubella” test system (Novosibirsk, Russia) according to the manufacturer’s instructions: IgG levels to rubella virus of 30 IU/mL and above were considered protective; results with IgG levels of 16–29 IU/mL were classified as negative. Samples with concentrations below 16 IU/mL of IgG to rubella virus were considered negative [19]. For mumps, optical density (OD) levels were determined, and results were considered positive when OD levels were >0.467. Empirically, mumps antibody levels were calculated based on OD values using the formula Index = OD sample/0.467, where OD sample is the optical density value [20]. Samples with an index ≥ 1.0 (i.e., OD > 0.467) were classified as seropositive. All mumps analyses were performed on the index scale. Varicella antibody levels were determined by solid-phase ELISA using the “Vector-Best IgG-varicella” test system (Novosibirsk, Russia) according to the manufacturer’s instructions: IgG levels to varicella > 1 IU/mL, and levels above that were considered protective; samples with concentrations < 1 IU/mL to varicella virus were considered negative [21]. Antibody values for measles, rubella, mumps, and varicella were logarithmically transformed to reduce variance and achieve a normal distribution.

Statistical analysis was performed using Microsoft Excel 2024 software package and RStudio software version 4.5.1 (R Foundation for Statistical Computing, Vienna, Austria). Normality of quantitative variables was assessed using the Shapiro-Wilk test. Quantitative data are presented as medians with interquartile range (IQR) for non-normal distributions. Qualitative data are presented as proportions (percentages). For quantitative data, statistical significance of differences between groups was assessed using the Mann–Whitney test, given non-normal distribution. For qualitative data, Pearson’s χ^2^ test was applied, with determination of odds ratios (OR) and 95% confidence intervals (95% CI). Differences in concentration between pediatric health groups were assessed using the Kruskal–Wallis test. Associations between ln-transformed concentrations and age or time since the last dose were assessed by simple linear regression. The relationship between quantitative and qualitative data was determined using logistic regression. Test results were considered statistically significant at *p* < 0.05.

## 3. Results

### 3.1. General Characteristics of the Study Group

The present study included 156 children. The demographic characteristics of the group were as follows: 83 (53.2%) girls and 73 (46.8%) boys. Study participants were divided into three age groups—children aged 13, 14, and 15 years, respectively. The distribution across groups was as follows: group 1–55 (35.3%) individuals; group 2–71 (45.5%) individuals; group 3–30 (19.2%) individuals. Additionally, study participants were classified by pediatric health status categories. Twenty-five (16%) participants belonged to health group I; 66 (42.3%) to group II; and 65 (41.7%) to group III.

### 3.2. Immune Status of Patients

**Figure 1:**
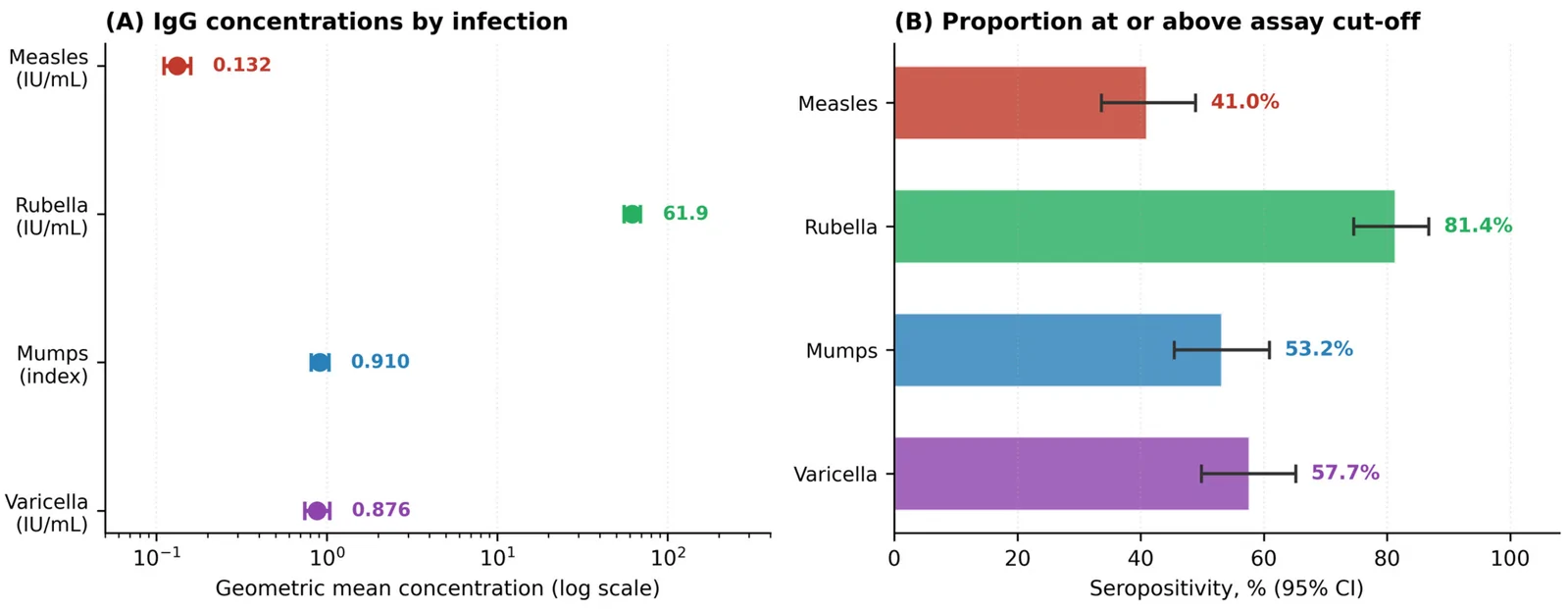
Humoral immunity profile against measles, rubella, mumps and varicella. Notes for Figure 1. Panel (**A**): geometric mean concentrations of IgG with 95% confidence intervals, plotted on a logarithmic scale. Because the four assays are calibrated on different scales (IU/mL for measles, rubella and varicella; a unitless index for mumps), concentrations are not directly comparable between infections and are shown for descriptive purposes only. Panel (**B**): proportion of participants with concentrations at or above the assay-specific cut-off, with 95% confidence intervals calculated by the Wilson method. Cut-offs: measles ≥ 0.18 IU/mL; rubella ≥ 30 IU/mL; mumps index ≥ 1.0; varicella ≥ 1.0 IU/mL.

All patients included in this study had complete vaccination against measles-rubella-mumps. Immunity against varicella was acquired through natural infection in childhood. The median age at varicella infection was 3 years (IQR 2–5). No severe cases or complications from the infection were recorded. Antibody concentrations varied widely between infections, and because the four assays are calibrated on different scales (IU/mL for measles, rubella and varicella; a unitless index for mumps), concentrations are not directly comparable across infections and are presented descriptively (Figure 1A). The lowest geometric mean concentration was observed for measles (0.132 IU/mL; 95% CI 0.111–0.158) and the highest for rubella (61.9 IU/mL; 95% CI 55.3–69.2), with mumps (0.910 OD; 95% CI 0.808–1.026) and varicella (0.876 IU/mL; 95% CI 0.741–1.036) intermediate on their respective scales. The proportion of participants with concentrations at or above the assay cut-off differed significantly between infections (χ^2^ 55.1; df = 3; *p* < 0.001) (Figure 1B). Seropositivity was highest for rubella at 81.4% (127/156; 95% CI 74.6–86.7), followed by varicella at 57.7% (90/156; 95% CI 49.8–65.2) and mumps at 53.2% (83/156; 95% CI 45.4–60.9). The lowest value was recorded for measles, at 41.0% (64/156; 95% CI 33.6–48.9), so that 59.0% of this fully vaccinated cohort had measles IgG below the applied cut-off.

### 3.3. Age and Immune Status

**Figure 2:**
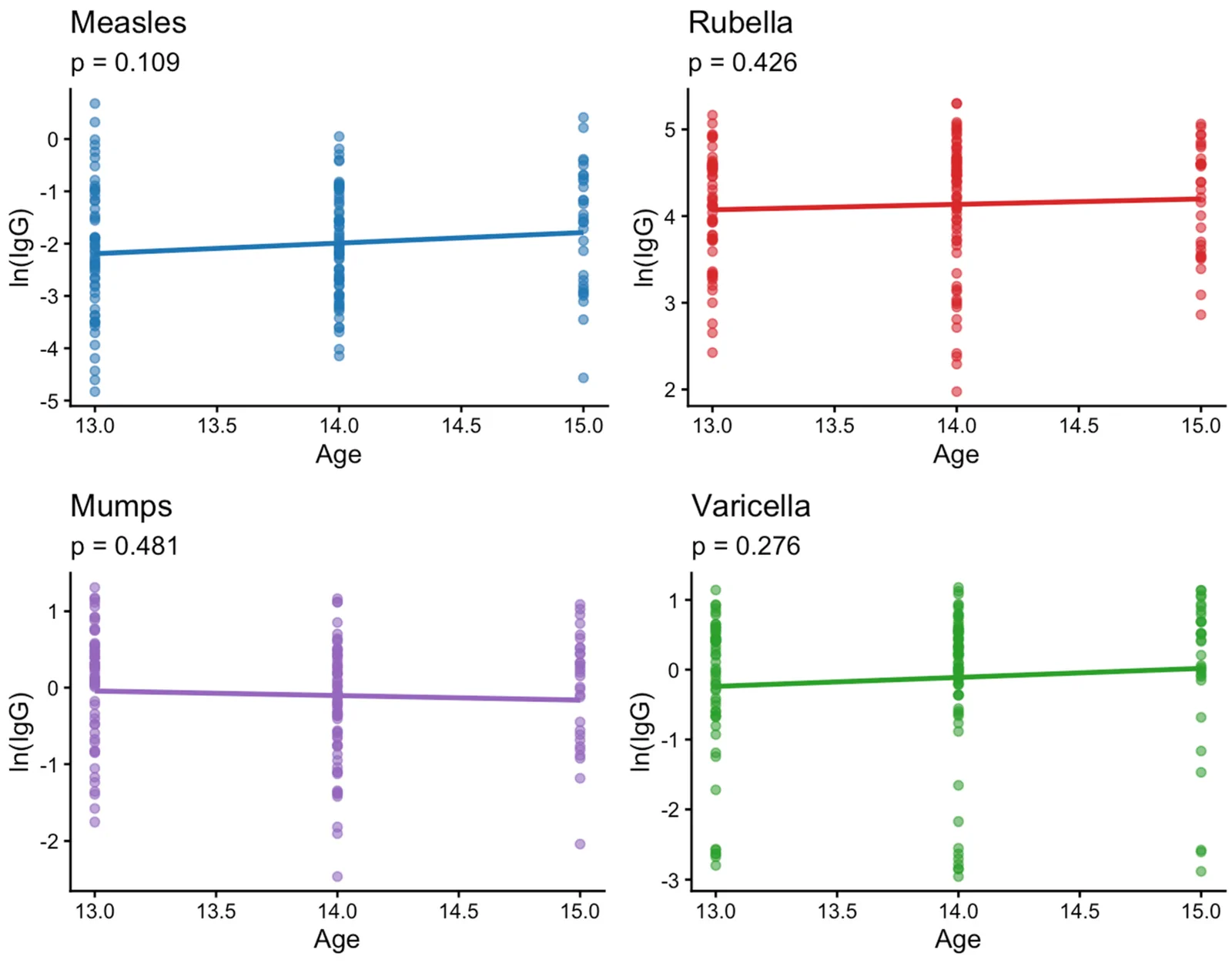
Antibody levels to measles, rubella, mumps, and varicella by age. Notes for Figure 2. Scatter plots with linear regression lines (solid); *p*-values correspond to the regression coefficient for age (continuous variable).

To assess potential waning of antibody levels during adolescence, linear regression analysis was performed to evaluate the relationship between age and logarithmically transformed antibody concentrations for each infection separately (Figure 2).

Analysis revealed a positive association between age and measles antibody concentrations, although it did not reach statistical significance (β 0.202; 95% CI −0.046–0.45; *p* = 0.109). In contrast to measles, rubella antibody concentrations remained stable across all age groups (β 0.063; 95% CI −0.093–0.218; *p* = 0.426). For mumps, a weak negative correlation was found between age and antibody concentration (β −0.06; 95% CI −0.226–0.107; *p* = 0.481). For varicella antibodies, a slight positive increase with age was detected (β 0.129; 95% CI −0.104–0.361; *p* = 0.276), indicating a minor trend toward increasing antibody concentrations with age. All four models explained less than 2% of the variance in antibody concentration (R^2^ ≤ 0.017). Given the three-year age range available, these analyses have limited power to detect gradual change, and the absence of a significant association should not be interpreted as evidence either of decline or of stability.

### 3.4. Relationship Between Pediatric Health Status Categories and Antibody Concentrations to Measles, Rubella, Mumps, and Varicella

**Figure 3:**
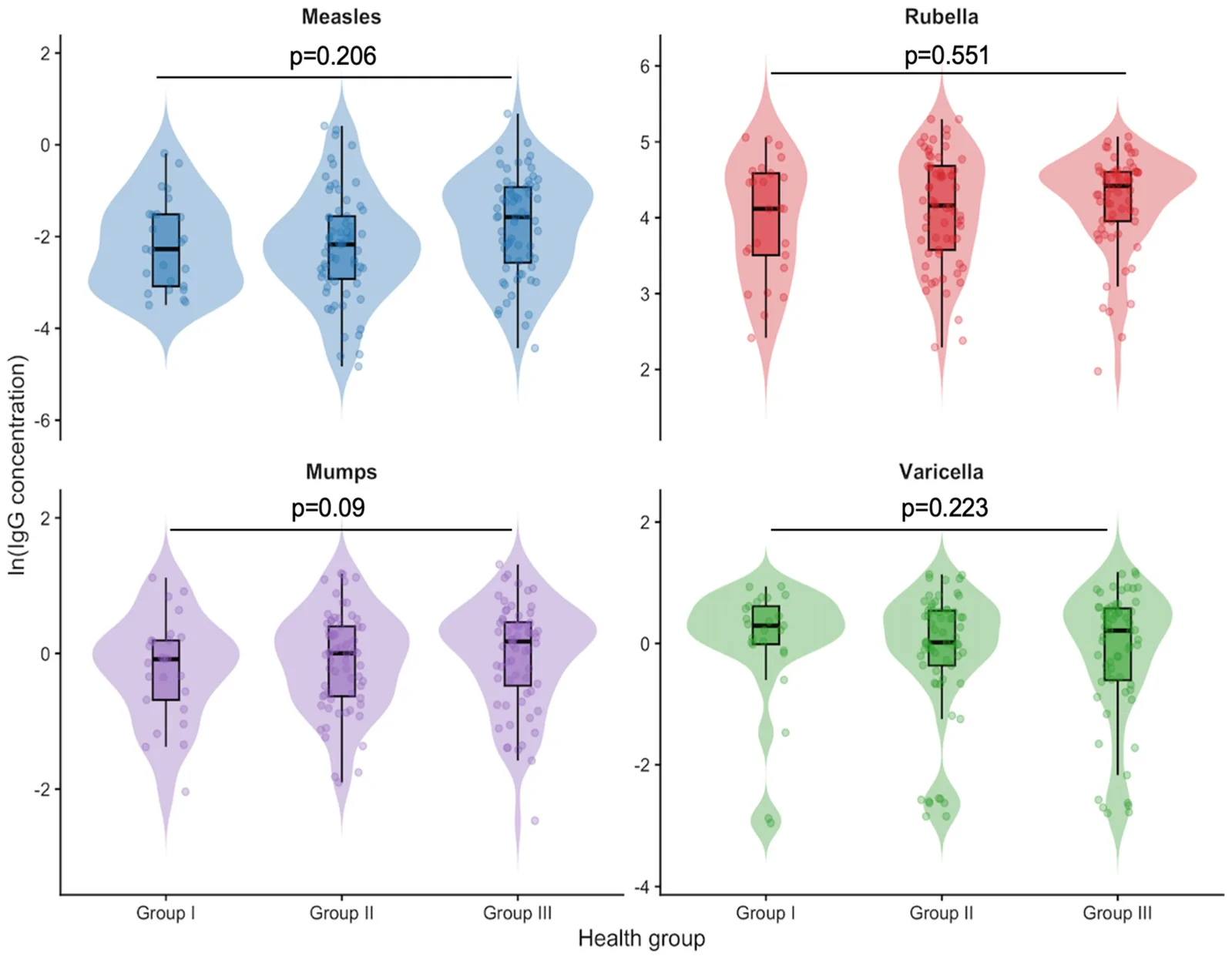
Distribution of antibody concentrations to measles, rubella, mumps, and varicella pediatric health status categories. Notes for Figure 3. The Kruskal–Wallis method was used to determine intergroup differences in children’s health status and logarithmic antibody concentrations.

Participants were divided into groups according to health status in accordance with standardized clinical criteria [17]. The distribution was as follows: group I (clinically healthy children without chronic diseases)—25 individuals; group II (minor functional abnormalities without chronic pathology)—66 participants; group III (chronic diseases in remission or compensated state)—65 individuals. Antibody concentrations demonstrated moderate variation between health groups (Figure 3).

Group III showed somewhat higher mean measles antibody concentrations (Me 0.213 (IQR 0.0624–0.465)) compared to groups I (Me 0.114 (IQR 0.0503–0.308)) and II (Me 0.124 (IQR 0.064–0.246)); however, no significant differences between groups were identified (χ^2^ 3.16; *p* = 0.206) (Figure 3). For rubella antibodies, a small gradient toward higher concentrations was observed in group II (Me 81.6 (IQR 42.6–105)), although the magnitude of the difference was small compared to within-group variability in group I (Me 62.4 (IQR 41.3–96.7)) and III (Me 77.3 (IQR 37.5–104)) (χ^2^ 1.19; *p* = 0.551). In group I (Me 1.23 (IQR 0.619–1.72)), slightly higher mumps antibody values were observed, but between-group differences with groups II (Me 0.868 (IQR 0.524–1.33)) and III (Me 1.19 (IQR 0.549–1.65)) were not significant (χ^2^ 4.81; *p* = 0.09). Varicella antibodies were higher in groups II (Me 1.24 (IQR 0.752–1.72)) and III (Me 1.51 (IQR 0.911–2.16)) compared to group I (Me 1.02 (IQR 0.534–1.62)), but no differences between groups were detected (χ^2^ 3.00; *p* = 0.223).

### 3.5. Relationship Between Time Elapsed Since Vaccination and Antibody Concentrations to Measles, Rubella, and Mumps

**Figure 4:**
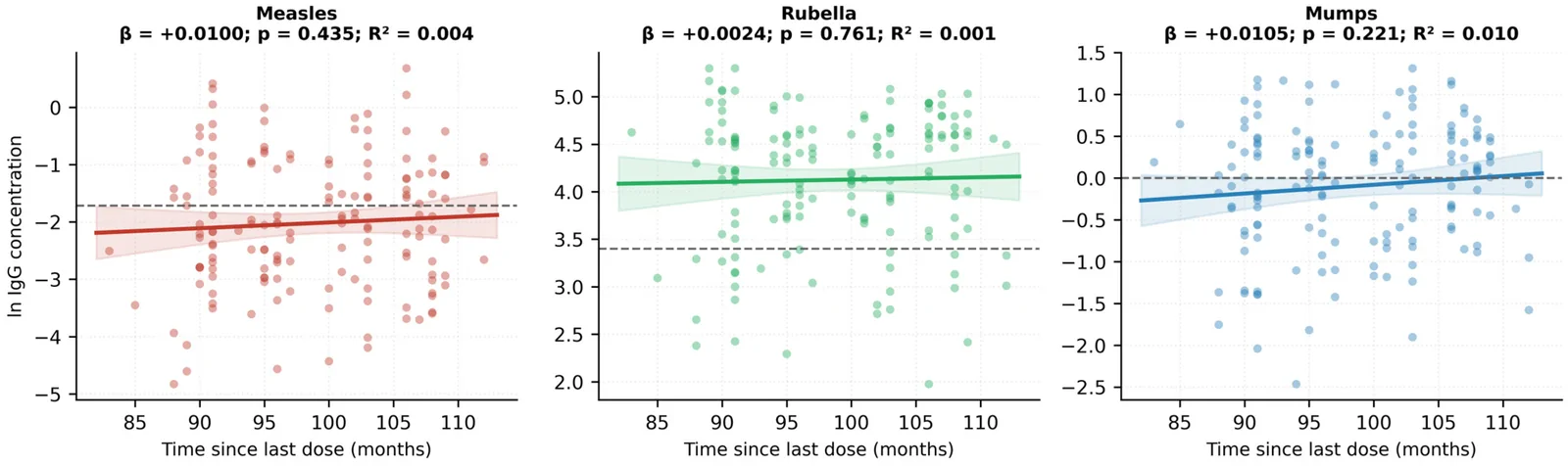
Relationship between time elapsed since last vaccination and antibody titers to measles, rubella, and mumps. Notes for Figure 4. Solid lines show simple linear regression with 95% confidence bands; dashed horizontal lines indicate the assay cut-off. β coefficients, *p*-values and R^2^ correspond to the regression coefficient for time since vaccination.

To assess potential decline in antibody levels, simple linear regression models were constructed separately for each infection (Figure 4).

The median interval between the last documented dose and blood sampling was 98.7 months (IQR 83–112 months). Linear regression analyses assessing the association between time elapsed since last vaccination and ln-transformed antibody concentrations revealed minimal explanatory power across all infections (R^2^ < 0.02 for all models, all *p* > 0.2). Specifically, time since vaccination accounted for less than 1% of variance for measles (R^2^ 0.004; *p* = 0.435), rubella (R^2^ 0.001; *p* = 0.761), and mumps (R^2^ 0.01; *p* = 0.221).

### 3.6. Sensitivity of Seropositivity Estimates to the Assay Cut-Off

Because the cut-offs applied in the primary analysis classified the manufacturers’ equivocal ranges as negative, the analysis was repeated with equivocal results classified as positive. For measles, 73 of 156 sera (46.8%) were below 0.12 IU/mL, 19 (12.2%) fell within the equivocal range 0.12–0.17 IU/mL, and 64 (41.0%) were ≥0.18 IU/mL; seropositivity was therefore 41.0% (95% CI 33.6–48.9) under the criterion applied here and 53.2% (95% CI 45.4–60.9) at the manufacturer’s cut-off of ≥0.12 IU/mL. For rubella, 8 sera (5.1%) were below 16 IU/mL, 21 (13.5%) fell within 16–29 IU/mL and 127 (81.4%) were ≥30 IU/mL, giving 81.4% and 94.9% (95% CI 90.2–97.4) respectively. The measles estimate thus shifted by 12.2 percentage points according to the handling of a single equivocal band.

## 4. Discussion

The present study provides a comprehensive assessment of long-term post-vaccination immunity to measles, rubella, and mumps, as well as post-infection immunity to varicella in adolescents aged 13–15 years. The obtained results demonstrate pronounced heterogeneity of immunological profiles depending on the infection and reveal critical gaps in population immunity.

Our study showed that only 41% of adolescents aged 13 to 15 years had detectable measles antibodies above the assay cut-off, which is significantly below the levels expected to ensure herd immunity and prevent transmission. The GMC of measles antibodies was also the lowest among all studied infections (GMC 0.132), indicating not only a low proportion of seropositive individuals but also relatively low antibody titers even among those formally exceeding the threshold antibody concentration. A 2020 meta-analysis showed that among individuals aged 18 to 30 years, the proportion seronegative for measles virus was 27.3% (95% CI 25.7–27.3%). Among children under 17 years who received two doses of measles vaccine, this proportion was significantly higher: 38.3% (95% CI 35.8–40.8%) [22]. These data are consistent with trends observed in other countries with high vaccination coverage. Studies in Scandinavia have shown that 5–10% of individuals vaccinated with two doses of MMR remain seronegative in adolescence and young adulthood [7]. Our previous study evaluating protective antibody titers after measles and mumps revaccination at age 6 or 7 years showed that 12 months after booster dose administration, seropositive individuals had low or moderate levels of measles virus antibodies, and the proportion of measles virus seronegative individuals was 32.5% [23]. However, we cannot state that upon exposure to “wild” measles antigen, seronegative individuals will necessarily develop this infectious disease, since live vaccines induce both humoral and cell-mediated immune responses [24]. However, it is known that circulating antibodies prevent virus entry into target cells, while cellular immunity is responsible for virus elimination. This means that a seronegative person may contract measles, but will have an “attenuated”, mildly symptomatic form of the infectious disease [25]. In 2020, Italian authors conducted studies comparing immune status in response to measles booster vaccination in a group of individuals previously vaccinated against measles and in those who reported previous infection. The authors reported that 10 years after revaccination, the proportion of individuals from the vaccinated group who did not have protective antibodies against measles was 20% (GMC 92.2 IU/mL), while among those who reported previous infection, the similar proportion was 6% (GMC 213.3 IU/mL) [26]. It is noteworthy that in our study, all adolescents received complete measles vaccination. This fact is critically important—even among fully vaccinated adolescents, circulating antibody concentrations were relatively low, potentially reflecting either suboptimal primary response or gradual antibody decline over time in this age group. Early primary vaccination (12–15 months) may coincide with the period of residual maternal antibody influence, which reduces vaccine immunogenicity. The observed distribution of antibody levels highlights a cohort with diminished circulating titers, underscoring the importance of ongoing epidemiological surveillance alongside national morbidity data [8].

Rubella showed the highest seropositivity and the highest antibody concentrations among the four infections studied, with 81.4% of participants (95% CI 74.6–86.7) at or above 30 IU/mL and a GMC of 61.9. These results are consistent with the biology of rubella virus and characteristics of the live attenuated vaccine. The rubella vaccine strain possesses high immunogenicity, inducing seroconversion in more than 95% of vaccinees after one dose [2]. Studies confirm long-term antibody persistence over several decades after vaccination without significant decline [2]. Nonetheless, 18.6% of this fully vaccinated cohort had concentrations below the 30 IU/mL threshold applied here, and 5.1% were below the manufacturer’s cut-off of 16 IU/mL; seropositivity was therefore high but not universal. Stable rubella immunity in adolescence provides protection during the reproductive period, which is critically important for preventing congenital rubella syndrome.

The proportion of individuals with detectable mumps antibodies was 53.2%. While interpretation is complicated by the absence of a universally established correlate of protection for mumps, this observation aligns with international data regarding post-vaccination antibody decline. This is consistent with international data showing that mumps demonstrates less temporally stable post-vaccination immunity among MMR vaccine components (compared to rubella) [27]. Protection provided by the mumps vaccine wanes on average 27 years after receiving two doses, ranging from 16 to 51 years [27]. Mumps outbreaks in populations with high vaccination coverage have been documented in the USA, Europe, and Asia [28]. Authors identified the following mechanisms contributing to increased incidence in both favorable and unfavorable epidemiological settings: primary vaccine failure (~5% of individuals do not respond even to two doses of MMR vaccine); secondary vaccine failure due to waning of specific antibodies; possible antigenic mismatch between vaccine strain and circulating virus genotypes [28]. Russian data show that twice-vaccinated individuals comprise 10.5% of mumps cases, which indirectly confirms the problem of secondary vaccine failure [8]. Our results demonstrating suboptimal seroprotection in adolescence explain the vulnerability of this population to outbreaks, especially in conditions of prolonged and close contact in organized settings (schools, colleges). It is important to note that even in the presence of mumps antibodies, protection may be insufficient. Currently, correlates of serological protection for mumps are not clearly defined compared to measles or rubella; the presence of post-vaccination IgG antibodies to mumps does not always guarantee disease prevention, especially with high viral load [29].

Separately, it is important to consider the specifics of measles, rubella, and mumps vaccination in Russia: routine vaccination against rubella, mumps, and measles is conducted at age 12 months, followed by revaccination at age 6 years [14]. Either monovalent rubella vaccine and bivalent measles-mumps vaccine or MMR vaccine is used. Study participants with protective levels of rubella antibodies were vaccinated against measles, mumps, and rubella with either a polyvalent vaccine or two different vaccines administered simultaneously. All participants had documented receipt of two doses of vaccine against MMR, administered according to the national schedule. Two mechanisms could contribute to the low seropositivity observed for measles and mumps: absent or inadequate initial seroconversion (primary vaccine failure) and decline of antibody after an initial response (secondary vaccine failure). Our data cannot distinguish between them. A single serum sample obtained several years after vaccination provides no information on the initial response, and in the absence of post-vaccination baseline titers neither mechanism can be excluded. It should also be noted that responses to the individual antigens of a combined vaccine are not necessarily concordant within an individual, so that seropositivity to one component cannot be taken as evidence that the response to another was adequate. Distinguishing these mechanisms would require prospective sampling from the time of vaccination, which we identify as a priority for further work.

Our study separately examined varicella antibodies. All study participants acquired humoral immunity through natural infection in early childhood. Particularly noteworthy is that only 58% of individuals maintained seropositivity based on the diagnostic threshold of varicella-zoster virus (VZV) antibodies. This is an unexpected result, considering that natural infection usually induces more stable immunity compared to vaccination. A possible explanation relates to VZV biology. After primary infection, the virus persists in a latent state in dorsal root ganglia [30]. Periodic subclinical reactivation or exposure to the virus from others may maintain or enhance humoral immunity (“natural boosting” phenomenon) [31,32]. However, in the absence of such exposure, antibodies may gradually decline, especially if circulating antibodies are not maintained by active plasma cell production [31,32]. The identified positive correlation between age and VZV antibody concentration (β = +0.129) may reflect the cumulative effect of subclinical reactivations or repeated virus contacts in adolescence. In conditions of high varicella circulation in Russia (more than 842,000 cases in 2025), adolescents have numerous opportunities for immunity boosting through contact with younger children. It is important to emphasize that the absence of varicella vaccination in Russia’s National Immunization Schedule is a “missed opportunity” (the introduction of the varicella vaccine into the National Immunization Schedule is planned for 2029) [14].

Contrary to expectations of progressive antibody decline to studied infections with increasing time after vaccination (measles, rubella, mumps), none of the studied infections demonstrated statistically significant age-related decline in the 13–15 years range. The narrow observation time window (3 years) may be insufficient to detect gradual waning, especially if it occurs at slower rates or has already concluded in an earlier period. Antibody dynamics after vaccination are typically characterized by biphasic decay: rapid decline in the first 1–3 years followed by a plateau maintained by long-lived bone marrow plasma cells [24]. The present study likely captures the plateau phase, during which antibody concentrations are relatively stable. It is worth noting separately that convincing data on the relationship between measles, rubella, and mumps antibody titers and time elapsed since last vaccination were not obtained. The absence of a statistically significant association between time elapsed since vaccination and antibody titers across the studied age bracket should be interpreted with caution. Rather than confirming genuine long-term stability, this finding likely reflects methodological limitations—specifically the relatively narrow age window of 13–15 years and the cross-sectional design—rather than an absence of gradual waning over longer intervals.

An important consideration in interpreting the low measles seropositivity observed here is the performance of IgG enzyme immunoassays relative to the plaque reduction neutralization test (PRNT), which remains the reference standard for measles serostatus. In a systematic review of head-to-head comparisons, Lutz et al. showed that commercial measles IgG EIAs are optimized for individual-level diagnosis and are consequently weighted toward specificity, so that they may classify as negative a substantial proportion of sera that are positive by PRNT [33]. The authors note that assays with inadequate sensitivity underestimate population immunity, and that the handling of equivocal results markedly affects measured sensitivity: grouping equivocal results with positives rather than negatives increased sensitivity by up to 35.3 percentage points [33]. Our own sensitivity analysis is consistent with this, with the measles estimate shifting from 41.0% to 53.2% according to the treatment of a single equivocal band. The estimate reported here should therefore be regarded as a conservative lower bound rather than as a measure of the proportion of susceptible individuals. Confirmation of a subset of sera by PRNT, or by IgG avidity testing, would be required to establish the extent of any true immunity gap.

### Limitations

Several limitations should be noted. First, the cross-sectional design cannot establish causal relationships or capture individual trajectories of antibody concentration, and no data on titers immediately after vaccination were available, so individual or population rates of decline and antibody half-lives could not be estimated. Second, the narrow age range (three years) and the limited range of intervals since the last dose (6.9–9.3 years) provide limited power to detect gradual change. Third, only circulating IgG was measured; cellular immunity, memory B cells and antibody avidity were not assessed, and seronegativity by ELISA therefore does not exclude immunological memory or the capacity for an anamnestic response. Fourth, all serostatus classifications depend on commercial enzyme immunoassays and on the cut-offs applied. As the sensitivity analysis shows, the measles estimate varies by more than 12 percentage points according to the treatment of equivocal results, and no confirmatory neutralization testing was performed. For varicella, the ≥1.0 IU/mL threshold is a diagnostic cut-off rather than a validated correlate of protection, and commercial VZV IgG ELISAs are recognized to have limited sensitivity relative to FAMA or gpELISA; the proportion classified as seronegative after natural infection is therefore likely to overestimate the true absence of immunity. Fifth, participants were recruited at a single site and were not sampled probabilistically; pediatric health status categories II and III were over-represented relative to the general adolescent population, and the findings should not be extrapolated nationally. Finally, prior varicella was established from medical records rather than by laboratory confirmation at the time of infection.

## 5. Conclusions

In this cross-sectional study of 156 adolescents with documented two-dose MMR vaccination, the proportion with IgG concentrations at or above the applied assay cut-offs differed markedly between infections. Seropositivity for measles and mumps was therefore substantially below what would be expected after two documented doses, whereas rubella showed both the highest seropositivity and the highest concentrations. The measles estimate proved strongly dependent on the treatment of equivocal results, rising to 53.2% under the manufacturer’s cut-off. Because a single measurement of circulating IgG does not capture cellular immunity or immunological memory, and because enzyme immunoassays are known to underestimate seroprevalence relative to neutralization testing, these findings describe the distribution of circulating antibody in this cohort rather than the prevalence of susceptibility to infection. They indicate that the durability of measles- and mumps-specific antibody responses in adolescents merits systematic investigation in larger, representative samples, with assessment of avidity, cell-mediated immunity and confirmatory neutralization testing, before conclusions can be drawn for immunization policy.

## Data Availability

The data presented in this study are available on request from the corresponding author. The data are not publicly available due to patient privacy concerns and institutional data protection policies.
